# A health intervention or a kitchen appliance? Household costs and benefits of a cleaner burning biomass-fuelled cookstove in Malawi

**DOI:** 10.1016/j.socscimed.2017.04.017

**Published:** 2017-06

**Authors:** Katie Cundale, Ranjeeta Thomas, Jullita Kenala Malava, Deborah Havens, Kevin Mortimer, Lesong Conteh

**Affiliations:** a Health Economics Group, School of Public Health, Imperial College London, St Mary's Hospital, Norfolk Pl, London W2 1PG, UK; b Medical Research Council Centre for Outbreak Analysis and Modelling, Department of Infectious Disease Epidemiology, School of Public Health, Faculty of Medicine (St Mary's Campus), Imperial College London, London, Norfolk Pl, London W2 1PG, UK; c Malawi Epidemiology and Intervention Research Unit (MEIRU), P.O. Box 46, Chilumba, Karonga District, Malawi; d Liverpool School of Tropical Medicine, Pembroke Place, Liverpool L3 5QA, UK; e Malawi Liverpool Wellcome Trust, Malawi, Queen Elizabeth Central Hospital College of Medicine, P.O. Box 30096, Chichiri, Blantyre, Malawi

**Keywords:** Malawi, Cookstoves, Qualitative, Economic costs, Time savings, Benefits

## Abstract

Pneumonia is the leading cause of mortality for children under five years in sub-Saharan Africa. Household air pollution has been found to increase risk of pneumonia, especially due to exposure from dirty burning biomass fuels. It has been suggested that advanced stoves, which burn fuel more efficiently and reduce smoke emissions, may help to reduce household air pollution in poor, rural settings.

This qualitative study aims to provide an insight into the household costs and perceived benefits from use of the stove in Malawi. It was conducted alongside The Cooking and Pneumonia Study (CAPS), the largest village cluster-level randomised controlled trial of an advanced combustion cookstove intervention to prevent pneumonia in children under five to date. In 2015, using 100 semi-structured interviews this study assessed household time use and perceptions of the stove from both control and intervention participants taking part in the CAPS trial in Chilumba. Household direct and indirect costs associated with the intervention were calculated.

Users overwhelming liked using the stove. The main reported benefits were reduced cooking times and reduced fuel consumption. In most interviews, the health benefits were not initially identified as advantages of the stove, although when prompted, respondents stated that reduced smoke emissions contributed to a reduction in respiratory symptoms. The cost of the stove was much higher than most respondents said they would be willing to pay.

The stoves were not primarily seen as health products. Perceptions of limited impact on health was subsequently supported by the CAPS trial data which showed no significant effect on pneumonia. While the findings are encouraging from the perspective of acceptability, without innovative financing mechanisms, general uptake and sustained use of the stove may not be possible in this setting. The findings also raise the question of whether the stoves should be marketed and championed as ‘health interventions’.

## Highlights

• The first evaluation of household perceptions of an advanced cookstove trial in Malawi.
• We investigate the intensive margin, i.e. how the technology is used and perceived.
• Significant benefits from reduced cooking and fuel collection times were reported.
• Unless prompted, respondents rarely associated the stoves with health benefits.
• The high cost of the advanced cookstove will need addressing to ensure future uptake.

## 1 Introduction

Around half of the world's population, mostly in low-income countries, relies on solid biomass fuels (such as dung, crop residues, firewood and charcoal) as their main means of cooking and heating fuel (WHO, 2013). These fuels are typically burned in open, usually three stone, fires which burn inefficiently, releasing numerous toxic partial products of combustion (Bruce et al., 2000, Ezzati and Kammen, 2002, Pant et al., 2014, Smith and Mehta, 2004). Household air pollution (HAP) released from the inefficient burning of solid biomass fuels has direct adverse impacts on human health, especially amongst young children and their mothers (Duflo et al., 2008, Gordon and Graham, 2006, WHO, 2013). Exposure to HAP has been found to nearly double the risk of pneumonia in children under five years of age (Dherani et al., 2008).

In an effort to reduce the negative health impacts of HAP among poorer households, and the negative externalities of biomass fuel consumption (including greenhouse gas emissions and deforestation), non-governmental organisations and governments have long been trying to disseminate cleaner burning cookstoves throughout much of Africa, Asia, and South America. The Global Alliance for Clean Cookstoves (GACC), an initiative undertaken by the United Nations Foundation, seeks to distribute 100 million clean cookstoves by 2020 (GACC, 2015).

The reported direct health benefits associated with clean cookstove use are varied. Studies have found that the reduced smoke emissions associated with cleaner burning cookstoves have led to health improvements (Clark et al., 2009) including reductions in respiratory symptoms (Alexander et al., 2014, Bautista et al., 2009, Burwen and Levine, 2012, Romieu et al., 2008) and a decrease in the incidence of acute lower respiratory infections (ALRI) (Ezzati and Kammen, 2002). However, other studies have found little or no evidence of health benefits (Hanna et al., 2012, Smith et al., 2011). Economic evaluations suggest that cleaner burning biomass-fuelled cookstoves are highly beneficial societal investments (García-Frapolli et al., 2010, Habermehl, 2007, Habermehl, 2008, Hutton et al., 2007, Mehta and Shahpar, 2004), although a modelling analysis suggests private net benefits may be negative, as the acceptability and use of cleaner stoves poses a challenge. More specifically learning how to use new stoves and adjusting to new fuels may be time consuming, inconvenient or culturally inappropriate (Jeuland and Pattanayak, 2012).

In spite of the efforts to promote their usage, advanced cookstove interventions have not seen widespread adoption and sustained use amongst households in low- and middle-income countries. Several reasons have been suggested (Lewis and Pattanayak, 2012, Rehfuess et al., 2014, Malla and Timilsina, 2014), such as the mixed evidence on the fuel consumption savings and health benefits discussed above, as well as the potential cost barriers and liquidity constraints which may drive the decision on whether or not to adopt cleaner stoves (Miller and Mobarak, 2015; Mobarak et al., 2012). More context-specific evaluations are therefore necessary to fully appraise the stoves in local circumstances and to understand the different aspects of adoption behaviour amongst households.

The health economics literature on adoption behaviour is an emerging area of research (see for example Bensch and Peters, 2015, Bensch et al., 2015, Dupas, 2011, Cohen and Dupas, 2010, Kremer and Miguel, 2007). In recent work, Dupas (2011) highlights the importance of including both the extensive margin of behaviour (mere adoption of a technology) as well as the intensive margin (how a technology is used and perceived) in evaluating the full effect of an intervention. Our study contributes to this literature by investigating the socioeconomic costs and benefits of adopting the new technology from the household's perspective using detailed primary data.

To our knowledge there are few qualitative studies that have examined the intensive margin of advanced combustion cookstoves, and certainly none in Malawi. The extent that the stoves are perceived as effective health products is discussed.

## 2 Methods

### 2.1 Study context

In Malawi, up to 95% of households rely on solid biomass fuels cooking (Fullerton et al., 2009): Pneumonia is the leading cause of under-five mortality in Malawi, with an estimated 1000 deaths in 2010 attributed to the disease (WHO, 2013). World Health Organization (WHO) guidelines on indoor air quality recommend maximum 24-h average air concentrations of no more than 35 mg/m^3^ PM_2.5_ (Bruce et al., 2015). In Malawi, however, a study into household air pollution found that within 80% of homes tested, PM_2.5_ levels were four times greater than the WHO level for outdoor air quality (Fullerton et al., 2009).

This qualitative study relates to The Cooking and Pneumonia Study (CAPS) (Mortimer et al., 2016). CAPS was a cluster-randomised controlled trial (RCT) undertaken in two sites in Malawi: Chikhwawa and Chilumba (Trial registration: ISRCTN59448623). The RCT aimed to understand if the provision of an advanced cookstove would prevent pneumonia in children under five years old. In 2012, a total of 100 village level clusters were randomised into control or intervention arms in Chilumba. Intervention participants were given two Philips HD4012 fan-assisted stoves, a solar panel to power the stoves, one cooking pot, user training, and maintenance support, in order to replace traditional cooking methods that use a three-stone fire. Training consisted of initial demonstrations at the community level and subsequent advice offered during scheduled three-monthly household visits. Damaged cookstoves were repaired and replaced as promptly as possible, acknowledging that there were inevitably brief periods when a household would be reliant on just one cookstove. As the Philips stove has a surface area for only one cooking pot at a time, participants were given two stoves to allow for users to cook multiple items at once to help minimise use of supplementary cooking methods (i.e. three stove fires). Engineered and manufactured as an “advanced” cookstove in Lesotho, the Philips stove reduces smoke emissions by up to 90% and has a thermal efficiency of up to 42% (SNV, 2013). Field tests in Chikhwawa suggested emissions associated with a given cooking task were reduced by approximately 75% compared to the open fire (Wathore et al., 2017). Control arm participants continued their usual cooking methods. Those in the control arm were sensitised to the trial at the same time as intervention participants and were told that they would receive two fan-assisted cookstoves at the end of the trial, on the grounds of equity, ethics and retention. Trial results, published in 2016, found no evidence that an intervention comprising cleaner burning biomass-fuelled cookstoves reduced the risk of pneumonia in young children in rural Malawi (Mortimer et al., 2016).

This qualitative study was conducted in the Chilumba CAPS trial site in 2015 when the trial results were unknown to both researchers and respondents. Chilumba is located in Karonga, a northern district of Malawi. The district is largely rural, with the approximate 270,000 person population relying mainly on subsistence farming and fishing (LSHTM, 2015a). The site is nested within the Karonga Prevention Study (KPS) research site which undertakes trials through villages registered in a demographic surveillance system – allowing researchers access to data collected in a sub-population of 35,000 since 1979 (LSHTM, 2015b).^42^ This was the first cookstove trial in the area. Prior to the study, there was no reported use of cleaner burning cookstoves in this setting.

### 2.2 Design and data collection

To align with the study design of CAPS, and to reduce the possibility of the Hawthorne Effect on intervention subjects (McCarney et al., 2007), participants were selected from both the control and intervention arms of the study. A sample size of 100 households was chosen to allow for a large sample for qualitative work. Using the CAPS participant database, ten village clusters were randomly selected, five from the control arm and five from the intervention arm. Ten households in each cluster were then randomly selected for interview.

An integrated approach to data collection and analysis was applied: a deductive organising framework was first established based on extant literature; and continuous, iterative analysis throughout data collection then allowed for the questions to change over time as themes most pertinent to the Malawian content were exposed.

Two, largely similar, semi-structured interview guides were created. Questions were designed to ascertain the costs, benefits (both health and non-health), and perceptions of the cookstove from those in the intervention arm, and to determine the perceptions of the cookstove from those in the control arm (See Annex 1). The primary outcome for the CAPS trial was measured in health benefits, however in this study we were keen to not assume the cookstove was perceived by households as primarily a ‘health product.’ We therefore structured the questionnaire such that there was an opportunity for respondents to initially respond to the benefits of the new stoves unprompted, and later in the interview we focused on health benefits. The interview guides were piloted and adjusted according to feedback from Malawian data collectors and a Malawian research scientist. The guide was written in English and translated to Tumbuka (the local language) by a data collector. A third party then back-translated the Tumbuka questions to English to ensure the translated questionnaire carried the intended meaning.

The guides contained both closed- and open-ended questions. The data collectors conducting the interviews were experienced KPS staff familiar with the study clusters. Training in qualitative research and feedback on interview technique was conducted during the piloting (one week) and throughout the data collection process. Two male data collectors were assigned to each interview. One led the interview with the household's primary cook and the other transcribed in Tumbuka. Interviews were transcribed as close to verbatim as possible. The transcription was then translated to English by two KPS data entry staff. Data was collected between the 3rd of July and the 7th of August 2015.

### 2.3 Data management and analysis

The data entry staff entered the translated interviews into formatted Excel files after each day of data collection. The short lag time between data collection and translation allowed for continuous analysis of the data. Analysis of the interviews was completed using the software package NVivo (Version 10.2.1 (1377) for Mac). Data was coded based on a deductive content analysis using four main themes: perceptions, usage, cost, and time. These parent themes were explored for additional themes and insights contained therein. Where appropriate, themes were analysed across intervention and control arms.

It is notoriously difficult to determine time usage and shadow prices (García-Frapolli et al., 2010, Bensch and Peters, 2015). Time savings were therefore based on reported differences prior to and after use of the Philips cookstove. The challenges of estimating and interpreting time use will be discussed later. In this study population, where respondents are largely engaged in informal, subsistence agriculture, if and how these largely domestic activities should be presented as formal income generating economic activities is contested. To reflect the different views in the literature we use three methods to shadow price time savings.

The first, as used in Bensch and Peters (2015), assumes that time saved cannot be converted to income generation. As time saved would be used only on domestic activities, and not income generation, this method does not result in a monetary benefit for time saved. A second method, as used in García-Frapolli et al. (2010), assumes that 25% of time saved can be converted to income generation. This method uses gross national income (GNI) per capita as a proxy for household wages. Finally, a third method, as used by Sicuri et al. (2012), assumes that 100% of time saved can be converted to labour wages. Using the minimum wage for rural Malawians, the time saved through use of the stove was multiplied by the hourly minimum wage.

The method used to elicit willingness to pay (WTP) was very basic: respondents were asked simply how much they would pay for one cookstove. While not in line with more sophisticated approaches usually used in economic evaluations (Cookson, 2003), the approach used here was chosen because of the formative nature of this qualitative study.

We use an August 2015 exchange rate of 487.5 Malawian Kwacha (MWK) = 1 US dollars ($) (FX Exchange Rate, 2015a). Costs incurred in US Dollars related to the purchasing of stoves and component parts were converted using the exchange rate of 1 US$ = 430 MWK, the rate at the time of purchase in January 2014 (FX Exchange Rate, 2015b). Stove costs were identified through study invoices and the stove manufactures.

### 2.4 Consent

Written informed consent was obtained at cluster and household-level (parent or guardian of child) prior to participation. Data collection, including qualitative interview questions, was covered under this consent. Ethical approval for the overall trial was granted by The Malawi College of Medicine Research Ethics Committee (Ref P.11/12/1308) and the Liverpool School of Tropical Medicine Research Ethics Committee (Ref 12.40) approved the protocol. Imperial College gave additional ethical clearance for this socio-economic study. Additional verbal consent was obtained at the beginning of each interview.

## 3 Results

One hundred interviews were conducted: 50 in each of the control and intervention arms of CAPS. Interviews lasted between 30 and 45 min. Averages, ranges, and figures shown are presented to provide an overview of respondents’ answers. Findings are presented under four main themes: (1) perceptions, (2) usage, (3) time, and (4) household costs and benefits. Supporting quotes from those in the intervention arm of CAPS study are coded as IR (Intervention Respondents) and for those in the control arm, CR (Control Respondents). Table 1 provides a summary of respondent characteristics.

**Table 1:** Socio-economic and demographic characteristics of participants.

Variable	Intervention (n = 50)	Control (n = 50)
	Percentage (unless otherwise stated)	
Age	36 years (18–66)	30 years (15–70)
Sex	Female: 88	Female: 96
	Male: 12	Male: 4
Minutes per day on Income Generation	256 min (60–660)	156 min (120–270)
Marital status	Married: 92	Married: 70
	Single: 2	In a relationship: 4
	Divorced: 4	Single: 2
	Widowed: 2	Divorced: 14
		Separated: 6
		Widowed: 2
		Other: 2
Educational level (respondent)	None: 0	None: 2
	Primary: 84	Primary: 62
	Secondary: 16	Secondary: 36
	Post secondary: 0	Post secondary: 0
Education level (household head)	None: 4	None: 0
	Primary: 54	Primary: 76
	Secondary: 34	Secondary: 22
	Post secondary: 4	Post secondary: 2
Occupation	Housewife: 82	Housewife: 72
	Farmer: 94	Farmer: 96
	Herding: 44	Herding: 48
	Gardening: 16	Gardening: 22
	Seller: 32	Seller: 52
	Shopkeeper: 8	Shopkeeper: 6
	Childcare: 84	Childcare: 94
	Domestic help: 94	Domestic help: 92
	Student: 4	Student: 4
Ownership	Radio: 50	Radio: 60
	Watch/clock: 8	Watch/clock: 20
	Bank account: 22	Bank account: 24
	Charcoal iron: 38	Charcoal iron: 34
	Sewing machine: 4	Sewing machine: 8
	Mobile phone: 72	Mobile phone: 82
	Mosquito net: 98	Mosquito net: 98
	Mattress: 62	Mattress: 68
	Bed: 76	Bed: 78
	Bicycle: 48	Bicycle: 48
	Canoe: 4	Canoe: 14
	Oxcart: 4	Oxcart: 14
Electricity	Yes: 10	Yes: 2
	No: 90	No: 98

### 3.1 Perceptions of the technology

#### 3.1.1 Advantages

The majority of IRs reported that they liked using the stove, and found the advantages to be that it cooked food quickly and used less fuel. See Table 2.

**Table 2:** Advantages identified by intervention and control respondents.

Advantage	Intervention	Control
	Number of respondents	
Saves firewood	43	21
Cooks fast	40	13
Less smoke	10	10
Reduces pneumonia	4	6
Good	2	3
Efficient	2	2
Feel superior using	2	
Less time preparing fuel	1	
Tasty food	1	1
No problem to light	1	
Looks nice	1	
No need to manually fan	1	
Less cough	1	3
Controllable flame		2
Portability		1
*Sub-total advantages*	*109*	*62*
Do not know of any advantages		18

Most CRs reported their knowledge of the stove came from friends who used the stoves. When CRs were asked what they perceived the advantages of the cookstove to be, most replied that it reduced the amount of firewood needed for cooking, and that the cookstoves cook food quickly, and produce little smoke. Eighteen respondents said they did not know of any advantages to the stove, either because they had not used one before or because they did not know anything about the cookstove. It is interesting to note that when asked this initial open-ended question (without steering the respondents to include health benefits) health improvement was only mentioned as an advantage in 8% of responses. In addition, reduction in pneumonia and coughs were more likely to be mentioned in the control arm than amongst those in the intervention arm who had experience of the advanced stoves.

Unprompted, IRs only mentioned perceived health benefits on five occasions out of a total of 109 noted advantages of cookstove use. Two respondents said:

IR: I only suggest, when I used open fire with firewood I felt much chest pains and pneumonia affected my children so much but now sicknesses have decreased.

IR: In the past, my child could often fall sick, since I received the stove, my child has stopped suffering.

When prompted to describe any health benefits associated with the stove, nearly half of IRs said that the health benefits were a reduction in cough and slightly less referred to a reduction in pneumonia. However, nearly one-in-five IRs responded that they did not know of any health benefit to the stove. Of the few respondents who specifically addressed the question of who in the household benefited from improved health in nearly all cases it was reported that ‘everyone benefited’.

Other perceived benefits included a reduction in general ‘illnesses’ (7 respondents) and less eye pain (5 respondents). Many people cited that illnesses were reduced because the stove produced less smoke, or linked the possibility that lower smoke emissions could be the reason for a reduction in illnesses:

IR: I don't fall sick frequently now, maybe the reason was smoke? I benefit a lot.

Two respondents in particular suggested that the reduction of childhood illness could lead to greater benefits for the overall household:

IR: There is a reduction of pneumonia and cough at my household. We have all been helped at this household because if a child falls sick, I will have problems and if I fall sick, my child will also have problems – who is then going to prepare food for the child?

IR: It helps save the children from pneumonia. All of us can benefit because it's us who care for the children.

When asked their perceptions, CRs largely said that health benefits associated with the stoves came from a reduction in coughs and pneumonia because the cookstoves produce less smoke. Other responses cited reductions in sneezing, TB, anaemia, and other diseases as potential health benefits of the cookstoves. Seven CRs said they were not aware of any health benefits associated with the intervention cookstove.

#### 3.1.2 Disadvantages

When asked about disadvantages, three-quarters of IRs reported there were no disadvantages to the cookstove. Those that did find problems with the stove were largely concerned about the future maintenance of the stove once CAPS finished and damage to the pots. Concerns were raised over where to get replacement parts and where to go for repairs. See Table 3.

**Table 3:** Disadvantages identified by intervention and control respondents.

Disadvantage	Intervention	Control
	Number of respondents	
No disadvantage	31	38
Future maintenance	4	2
Damage to pots	3	5
Solar panel not durable	2	
Small firewood pieces necessary	1	
Stove often damaged	1	2
Understanding how to use		5

There were two negative comments about the durability of the solar panels. Those who had problems with the panels often responded quite strongly:

IR: The solar panel doesn't help us, it is faulty time and again.

IR: This solar panel is nothing. It's not durable. It has gone for repairing several times.

As with the IRs, the majority of CRs did not identify any disadvantages. The few potential disadvantages flagged by the CRs included not understanding how to use the stove and damage to pots.

### 3.2 Cookstove usage: complement or substitute to traditional cooking methods

When asked whether they used any other methods of cooking apart from the intervention cookstove, 20 out of 50 IRs said that they used no other cooking methods. Most other respondents said that they used three stone fires when their cookstoves were damaged, had run out of power, or when cooking for larger gatherings. Additionally, a few respondents said that when their children were cooking, they used three stone fires:

IR: We use fire with three stands … when the children are alone at home because the base of the stove is made of plastic material so it can catch fire and cause accidents to children, so we forbid them [from using the cookstove] when we're out for farming.

Some respondents said that *nsima*, the local staple, tasted better when cooked on the advanced combustion cookstove. Respondents also reverted back to traditional methods to cook other particular types of food:

IR: Sometimes when I roast meat I use firewood with stands, I fear that the fat would enter into the stove when roasting.

IR: If I want to cook rice, I use on three supported stands because on stove, rice is not cooked properly.

### 3.3 Household time

**Fig. 1:**
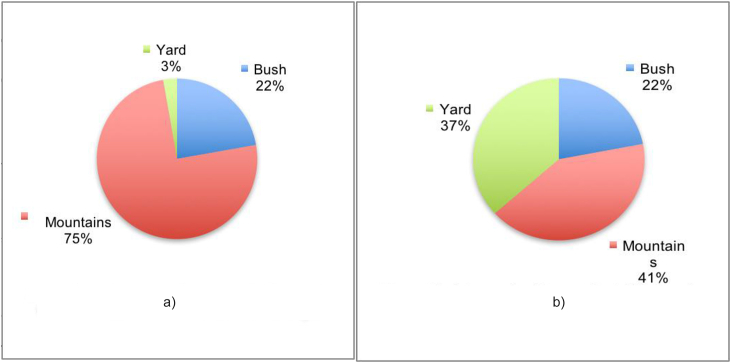
Intervention respondents' firewood collection sources before and after cookstove usage.

Prior to the intervention, a quarter of Intervention Respondents paid for fuel, while others collected firewood from the surrounding mountains, bushes, or household yards. Only 18% reported purchasing fuel when using the cookstove with the remaining reported using sources closer to their homes (see Fig. 1).

IR: We just fetch around the yard, we have stopped going up hills.

About three quarters of IRs (38 of 50) said that they had more time to do other things as a result of the time saved using the intervention cookstove – which included time collecting firewood and preparing and cooking food (Table 4). When asked what other things the respondent or other people in the household did with that time, all respondents said household chores.

IR: Washing while relish is being prepared, sweeping in the house while relish is being prepared.

**Table 4:** Reported times spent on cooking related Activities.

Activity	Control Respondents (CR)	Average (weekly) Reported time in hours (with range)		
		Intervention Respondents (IR)		
	No cookstove	Prior to cookstove	Using cookstove	Time saved
Collecting firewood	3.24 (0.25–15)	4.45 (0.41–16)	1.64 (0–9)	2.8 (0.42–7.0)
	Median: 2.3	Median: 4	Median: 1	Median: 3
Preparing	2.50 (0.35–21)	3.20 (0.35–21)	1.56 (0–15.65)	1.65 (0.35–5.25)
	Median: 1.75	Median: 1.75	Median: 0.7	Median: 1.05
Cooking	25.9 (1.98–70.0)	25.7 (4.67–56.0)	12.9 (1.75–31.5)	12.8 (2.92–24.5)
	Median: 21	Median: 21	Median: 11.4	Median: 9.63

Although the interviews asked who, in general, benefitted from the saved time, there was no mention of household members other than the primary cook benefiting from time saved.

### 3.4 Household costs & benefits

#### 3.4.1 Costs and willingness to pay

Table 5 provides a summary of the intervention costs. The cost of a Philips stove was 31,687.50 MWK ($65). If each of the households were to pay for the intervention and support provided by CAPS at the market rate paid by the study, the overall household costs for the purchase and use of two stoves, one solar panel, and one cooking pot, with user training and maintenance for one year, would amount to about US$227 or MWK110, 599.

**Table 5:** Household costs for advanced combustion cookstoves.^a^

Item/Activity	Quantity	Unit Cost (MWK)	Unit Cost ($)	Total (MKW)	Total ($)	Source/Assumption
*Direct (Financial)*						
Cookstove	2	31,687.5	65.00	63,375	130.00	ACE/CAPS
Delivery	2	17,062.5	35.00	34,125	70.00	ACE/CAPS
Solar panel	1	8287.5	17.00	8287.5	17.00	ACE/CAPS
Cooking pot	1	3500.0	7.18	3500.0	7.18	CAPS
Repair/Maintenance	2	656.0	1.34	1312	2.69	Repair twice a year based on informal conversations with those responsible for CAPS stove maintenance and interview responses
						Equates to 2 days salary at hourly wage of MWK82
**Total**				**110,599.5**	**226.87**	

a Costs based in August 2015.

**Fig. 2:**
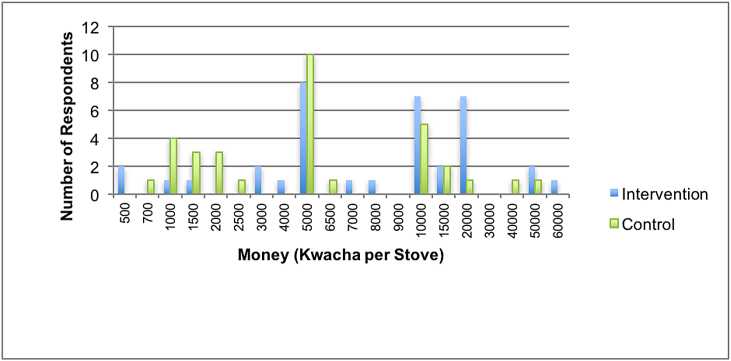
Number of respondents and amount willing to pay for one cookstove.

On being asked about their willingness to purchase the cookstove, all but two IRs said that they would purchase the cookstove were it available in shops. The average amount IRs said they were willing to pay was MWK12,700 (US$26.06). In contrast, thirty-nine CRs said that they would be willing to pay for the cookstove were it available in shops. The average amount CRs said they were willing to pay was MWK8,006 (US$16.42). The median amount given for both arms of the trial was MWK 5000(US$10.3). Fig. 2 shows the range and total number of respondents willing to pay certain Kwacha amounts per stove.

When asked how they determined the price they would pay for a cookstove, the majority of IRs replied that it seemed expensive and the amount they gave was all that they could afford. A few people also suggested that they could not determine the price because it would be enforced by the provider:

IR: Depending upon the price, but this can be negotiated, because a person cannot give his or her own cost price.

IR: We can talk at the amount they can charge, it's difficult to tell the exact price but I'd love to buy.

CRs gave similar answers, with the majority citing that the stove “seemed” expensive, and the amount given was all that they could afford. Furthermore, some respondents said that the stove was expensive because of the solar panels or because they had been told (by KPS staff) that the stove was expensive. One respondent, who gave MWK700 ($1.44), said:

CRs: Because they are there to help people in the villages, that's why they cannot be expensive.

#### 3.4.2 Benefits

Overall, the main benefits resulting from the use of an intervention cookstove amongst Malawian households are through time savings achieved on the basic household tasks of fuel collection and cooking, see Table 4.

Table 6 presents three interpretations of the financial gain from the time savings. Depending on the approach to shadow pricing outlined in the methods section, the benefits range from $0 to $127 per year. As all respondents cited time saved was used in domestic activities, and not income generation, the first method does not result in a monetary benefit for time saved. The second method assumes that 25% of time saved can be converted to income generation based on GNI. Using Malawi's GNI per capita MWK170, 625 (US$350) in 2015 (World Bank, 2015) and an additional 224 hours of work time, use of the cookstove could result in an increase of MWK18, 383 ($37.7) in household income per year. The third method assumes that 100% of time saved can be converted to domestic wages. The minimum wage in Malawi was raised to MWK551 per day in 2014 (Wage Indicator, 2014), or MWK68.875 per hour ($0.14). Therefore, a total of 897 hours saved annually could result in an additional MWK61, 764 ($127) earned in income per year.

**Table 6:** Household time and shadow prices from use of the advanced combustion cookstove.

Indirect (Time)							
Activity	Average hours saved per year	Shadow price					
		MWK			USD		
		No time conversion	25% time converted	100% time converted	No conversion	25% converted	100% converted
Fuel collection	146	0	2, 993	10, 056	0	6.14	20.63
Preparation	86	0	1, 763	5, 923	0	3.62	12.15
Cooking	666	0	13, 653	45, 871	0	28.0	94.09
Training	−1.25	0	−25.625	- 86.1	0	−0.053	−0.177
**Sub total**	**896.75**	**0**	**18, 383**	**61, 764**	**0**	**37.7**	**126.7**

Cost calculations in Table 5 suggest the intervention cost $227 per household, with stoves and pots lasting between 2 and 5 years depending on intensity of use and attention to repair and maintenance. Compare this to a possible indirect benefit of up to $127 (Table 6) per year in non-health related benefits. If the CAPS trial had shown there to be an effect on pneumonia we would also be able to present here possible direct health care savings. During interviews, a minority of households reported a decrease in household symptoms of common smoke-related illnesses with use of the stove. These perceptions were supported by clinical trial data which showed that the effect on pneumonia was non-significant.

## 4 Discussion

### 4.1 Perceptions of use

Respondents who had received the stoves as part of the trial overwhelmingly stated that they liked using them, would continue using them after CAPS ended, and would purchase one if it were available in shops. Both those in the intervention and control arms of the study stated the firewood savings and fast cooking time of the stove to be the main advantages, with smoke reductions and reductions in household illnesses also identified as minor advantages. This, despite the explicit mention of health and specific health benefits of interest clearly stated on the CAPS trial information and consent forms. For example, the forms stated *‘Advanced cookstoves may have health benefits but we do not know …. We are doing this trial to find out whether an advanced cookstove has health benefits and particularly whether it reduces pneumonias in young children …. In addition to information about pneumonias in children, we will also collect information about respiratory symptoms and burns’*.

These findings align with current literature on what people value in new cooking technologies. Only 8% of respondents identified health improvements as an advantage to the stove unprompted, although most respondents could identify health benefits associated with the stove when asked directly. The emphasis on non-health considerations such as fuel and time savings as the main benefits to the stove echoes previous literature. Mobarak et al. (2012) found that rural Bangladeshi women valued the ability of non-traditional cookstoves to reduce fuel use the most, with the second most valuable attribute being the ability to reduce cooking time. Previous publications considering the acceptability, performance, and use of the Philips cookstove in particular found similar time savings, fuel savings, and smoke reductions as benefits of the stove (Hegarty, 2006, Mukhopadhyay et al., 2012, SNV, 2013). More recently a trial in Peru of a home-based intervention package that included a cookstove was conducted on child health outcomes. While the trial showed no impact on respiratory health the “Convenience gains from improved cooking stoves and kitchen sinks [we]re highly valued by the beneficiaries” (Hartinger et al., 2016).

### 4.2 Cost to the household

The biggest contributor to household costs for this intervention would be the purchasing and maintenance of the stove and replacement of damaged costs. From a household perspective, the current cost of two stoves, one solar panel, one cooking pot, and maintenance, would be $227 (MWK110, 599). To date, the only evaluation of an advanced combustion stove in Malawi focused on the economic benefits (not accounting for health gains) for institutional Rocket Stove users, along with the benefits derived from environmental impacts (Habermehl, 2008). Habermehl (2008) found that an investment of US$1 gave a return of US$5.16 when economic benefits relating to reductions in fuel costs, preservation of forest reserves, and reductions in greenhouse gas emissions were considered.

As the stove was commercially produced in Lesotho, one option to reduce costs could be to promote locally produced stoves that could utilise regional materials to reduce transportation and unit costs, as well as engage the Malawian public in manufacturing jobs. However, as setting up a production factory is in Malawi is an unlikely short-term solution, alternative financing mechanisms should be considered. Some programmes have found that subsidised stoves, or long-term payment plans, have helped households invest in advanced cookstove technologies (Bailis et al., 2009, Debbi et al., 2014, Kshirsagar and Kalamkar, 2013). However, other programmes found that subsidising stoves may be an issue if the population does not value the product (Slaski and Thurber, 2009), if the product is provided free of charge (Adrianzén, 2011), or if the funding bodies shift priorities or run out of money (Bailis et al., 2009). Given that the target population for these advanced stoves is among the poorest in the world, any chosen financing method needs to ensure it is affordable to all Malawians. As one respondent said, *‘ … the technology is designed to help poor communities, and therefore should be available at an affordable price for rural Malawians’.*

As it stands, the actual cost of the stove is too high in comparison to what most respondents said they would be willing to pay for it. Although not a formal WTP analysis, the findings give an idea of the range of prices Malawians might consider. With costs at $227 for two stoves, it seems unlikely that direct purchase of the stove is appropriate in a rural Malawian context. With the average WTP amount being $26 (IRs) and $16.4 (CRs), it seems highly improbable that rural Malawians would be able to afford one cookstove, let alone two. Note the gross national income per capita in Malawi is just $350 (World Bank, 2015). As can be seen from the difference between CRs and IRs WTP, those who have used the cookstove place a higher price on its worth. Members of the community not experienced with the stove may therefore value the stove much less, and could be discouraged from purchasing the stove in the future unless community information sessions are established.

A question regarding use of the solar panels was included in the interview guide because CAPS staff members reported high rates of users tampering with the solar panels. By re-wiring the solar panels, users are able to power radios, lights, and charge phones. In a context where access to electricity is severely limited, it is both ingenious and understandable that users would want to use the panels for other means. Expanded training on how to use the stove and the component parts could decrease the need for maintenance, and therefore decrease the potential expenditures of the households.

### 4.3 Strengths and limitations

Open-ended responses in the semi-structured interview were expected, *a priori*, to contain enough variety and detail to enable qualitative coding of many themes to compare and contrast across respondents. However, in most interviews, women gave very short, to the point, answers. The succinct answers made it near impossible to pick up any subtle differences. Although the data collectors were encouraged to probe further and leave the questions open-ended, responses remained limited and structured. The data collectors suggested that perhaps the women did not have more to say on the subject of cookstoves, and the use of other methods – such as Focus Group Discussions – might not necessarily illicit more depth and richness of responses. To gain an understanding of the wider social and cultural factors that may facilitate sustained use of cleaner burning biomass-fuelled cookstoves a Photovoice approach was piloted at the other CAPS site (Ardrey et al., 2016). This used a photographic technique that allows people to share their perspectives and priorities through the photos they take and the reflections they share. While the emphasis of the Photovoice study was to explore the methodology, sociocultural, economic and health domains were highlighted as important determinants of use as was the role of gender.

Although many participants, of both the control and intervention arms, did not initially mention health benefits as advantages to the stove, the few that did could have done so purely because they were told that stove usage hoped to reduce pneumonia when recruited to the clinical trial, as mentioned above. Furthermore, as participants were given the cookstoves for free at the beginning of the trial, or were expecting them for free at the end, this could have led to what they perceived as “acceptable” responses for fear that the cookstove may be taken away. However, as some respondents did provide negative feedback and the health benefits reported certainly did not routinely echo the information sheet and consent form, we feel reassured that bias was not prevalent, and all respondents were assured at the beginning of each interview that answers would be anonymous and not affect cookstove ownership. Finally, participants in CAPS were also told the price of the stove, which could have influenced WTP prices given.

The precision of reporting time spent on activities under interview conditions is contested and should be interpreted with caution. However, given the consistency of the reported time estimations between IRs prior to using the cookstove and CRs using three stone fires, it is believed that the estimates for time saved reflect the reality, or was at least consistently bias across both groups of respondents.

As the majority of respondents did not spend money purchasing fuel, shadow pricing was used to proxy the benefits of time saved. Shadow pricing has been debated in economic evaluations of cookstove interventions (Bensch and Peters, 2015, García-Frapolli et al., 2010). Because rural populations have limited access to labour markets, and are instead involved largely in agricultural work, it is difficult to determine wages or time spent on income generating activities. Furthermore, time saved cannot be assumed to convert directly into potential economic activities. As was evident in the results of this study, the time saved through use of the cookstove was spent on domestic activities, rather than further income generation. Therefore, any formal conversion of time saved to income generation has a number of limitations, which is why we presented three methods of evaluating time saved. Nevertheless, the results from this study add context to the numbers. This is especially important for economic evaluations, as it is notoriously difficult to determine time usage and shadow prices (Adam et al., 2003).

Finally, while our findings on the perceptions of using cookstoves echo previous studies, future studies in more regions and other countries, especially ones with differing climates, fuel resources, types of cleaner burning biomass-fuelled cookstoves and poverty levels are needed.

### 4.4 Adoption and use

Previous studies into cleaner burning biomass-fuelled cookstoves mention numerous barriers to adoption, including financial costs, local acceptability, maintenance of the stoves, difficulty of use, low levels of formal education, gender dynamics in decision-making, and lack of knowledge as to the benefits of advanced stoves (Mobarak et al., 2012). As mentioned, the financial cost of the advanced cookstove would likely be the largest barrier to uptake in the future, along with the need to ensure continued maintenance is available to the local population. Local acceptability of the stove appears to be realised in this setting. The majority of respondents said that they (the women cooks) made the household decisions regarding purchasing of cooking equipment, however the issue of whether or not the husband or male household head would or could overrule the decision to purchase such a stove was not explored in this study. Understanding gender dynamics will therefore assist future uptake of the stoves in this context.

Continued use of a well-maintained stove is essential for households to fully reap the benefits of the intervention. Previous studies have found that usage (and therefore health impacts) declined over time because of poor maintenance of the stove (Hanna et al., 2012). As future maintenance of the stove was one of the largest themes found here, further training on household maintenance of the stove could aid in the reduction of damaged cookstoves.

## 5 Conclusion

Advanced cookstove usage has not been previously evaluated in Malawi, and therefore this study adds to the literature by describing the household perceptions of an advanced combustion stove in rural Malawi. The qualitative findings of this formative study show that the rural residents of Chilumba are both interested in use of the stove and find significant benefits from reduced cooking and fuel collection times. The health benefits of the stoves were not commonly identified by respondents. At the time of this qualitative study, the question remained unanswered as to whether the advanced stove would prevent, among other illnesses, pneumonia, thereby decreasing household costs associated with treating pneumonia in children under five. The CAPS trial has since found no evidence of a reduction in pneumonia in young Malawian children. In light of the trial findings it has been suggested that the lack of effect on pneumonia might be explained by exposure to additional sources of air pollution. Any attempt, therefore, to focus on a single source of air pollution exposure, such as the choice of cooking stove, is unlikely to be effective for improving health. To deliver health benefits, it may be that a more holistic and integrated approach to achieving clean air that tackles rubbish disposal, tobacco smoking, and other exposures, as well as robust cleaner cooking solutions (e.g., cleaner stoves and fuels) is needed (Mortimer et al., 2016, WHO, 2017). That said, while the financial cost of the stove is a concern given the low-income of the respondents, the acceptability and benefits from the stove suggest that these Malawians, and many others, could benefit from the continued use of this advanced combustion cookstove if appropriate financing mechanisms are established.

## Appendix A Supplementary data

Annex Annex
