# Supplementary material for: A health intervention or a kitchen appliance? Household costs and benefits of a cleaner burning biomass-fuelled cookstove in Malawi
Source: Soc Sci Med. 2017 Jun;183:1–10. doi: 10.1016/j.socscimed.2017.04.017 (PMC5446311; doi:10.1016/j.socscimed.2017.04.017)
Supplement: Annex [file mmc1.docx]

1. **VERSION OF THE QUESTIONNAIRE FOR THOSE IN THE CONTROL ARM**

**Prior to In-depth interview - Background Information**

**DEMOGRAPHIC DATA**

1. CAPS House Identity Number |__|__|__|__|-|__|__|__|-|__|__|
2. Date of Birth of respondent (dd / mm / yyyy) |__|__|-|__|__|-­|__|__|__|__|
3. Sex (**1**=Male **2**=Female) |__|
4. Husband present during the interview

Yes:

No:

**SOCIO ECONOMIC DATA**

| 1. Religion 2. Christianity 3. Islam 4. Traditional African Religion 5. None 6. Other___________________ | (cross one) | 1. Marital Status 2. Married 3. In relationship but not married 4. Single 5. Divorced 6. Separated 7. Widowed 8. Other __________________ | *(cross one)* |
| --- | --- | --- | --- |
| 1. How are you related to the household head?      1. I am the household head 2. My husband 3. My father 4. My father in law 5. My eldest brother 6. The eldest brother of my husband 7. The eldest brother of my father in law 8. My mother 9. Other (Specify) *___________* | *(cross one)* | 1. What is your Occupation 2. Housewife 3. Traditional Birth Assistant 4. Nurse/ Midwife 5. Farmer 6. Herding 7. Gardening 8. Sell at the market/street 9. Shopkeeper/ Retail 10. Childcare 11. Domestic Help 12. Student 13. No occupation 14. Other(Specify)___________ | *(cross all that apply)* |
| 1. What is the Occupation of your husband?      1. Imam 2. Marabout 3. Village health worker 4. Farmer 5. Herdsman 6. Fisherman 7. Bricklayer 8. Carpenter 9. Welder 10. Sell at the market/street 11. Shopkeeper/ Retail 12. Retired 13. No Occupation 14. Not applicable 15. Other (Specify) ___________ | *(cross all that apply)* | 1. What is the Occupation of the household head (if it is not you or your husband)      1. Imam 2. Marabout 3. Village health worker 4. Farmer 5. Herdsman 6. Fisherman 7. Sell at the market/street 8. Shopkeeper/ Retail 9. Retired 10. No Occupation 11. Not applicable 12. Other (Specify) _________ | *(cross all that apply)* |
| 1. Do you live under the same roof as your husband? 2. Yes *(Skip to Q13)* 3. No 4. N/A | | | *(cross one)* |
| 1. If you **do not** live under the same roof as your husband…..   Do **you own at least 1 of** the following?   1. Radio/cassette 2. Iron bed or carved wood bed 3. Bicycle 4. Horse or donkey cart 5. Boat 6. Video 7. TV 8. Motorbike 9. Watch 10. Mobile phones 11. Car 12. None of the above | *(cross all that apply)* | 1. In other households in **your compound** does anyone own the following? 2. Radio/cassette 3. Iron bed or carved wood bed 4. Bicycle 5. Horse or donkey cart 6. Boat 7. Video 8. TV 9. Motorbike 10. Watch 11. Mobile phones 12. Car 13. None of above | *(cross all that apply)* |
| 1. What is the main source of drinking water in your household 2. Surface water 3. Tanker 4. Well 5. Bore hole 6. Piped water 7. River water 8. Other _____________ | *(cross one)* | 1. What kind of toilet facilities does your household use? 2. No facility 3. Pit latrine 4. Improved pit latrine 5. Flush toilet 6. Other __________________ | *(cross all that apply)* |
| 1. In **your** household does anyone own the following? 2. Goats 3. Sheep 4. Horses 5. Donkey 6. Pigs 7. 0-50 Cattle 8. 51-100 Cattle 9. 101-200 Cattle 10. None of the above | *(cross all that apply)* |  |  |
| 1. What is the main material of the floor? 2. Earth/sand 3. Wooden/bamboo 4. Vinyl/tiles 5. Cement 6. Other _____________________ | (Cross one) | 1. What is the main material of the roof? 2. Plastic/tarpaulin 3. Thatch 4. Iron Sheets or tiles 5. Other ________________ | (cross one) |

**Semi-structured Questionnaire: Cookstove Usage**

**Introduction to study:**

*Good morning/afternoon, thank you for agreeing to participate in our survey. I’ll be asking you questions on your experience of the CAPS cookstoves so far – we are trying to better understand the costs and benefits of the stove from the users perspectives. We are here to learn from you and none of your answers will affect your place in the study.*

*I will be asking you questions about your experience cooking before and after the cookstove, regarding your time spent collecting fuel, cooking itself, and any impacts the stove has had on your lives since you started using it. Some questions will be yes or no questions, but for others, I encourage you to give as much detail as possible about your experiences.*

**Before Receiving the Study Cookstove**

*(*Preamble: *This set of questions will be focused on what you did BEFORE you started using the cookstove for this study)*

1. Before you received new cookstoves as part of this study, what sort of fuel did you use to prepare meals?
2. How long did you spend in total on cooking using this previous method each day?
3. Let us break this down,
   1. in the morning:
   2. in the afternoon:
   3. in the evening:

***Before you even start cooking, there is the process to fetching and maybe preparing the fuel:***

1. Were you involved in fetching and preparing the fuel for the cooking?

*(Preparing means: chopping the wood into smaller pieces, collecting charcoal, etc)*

Yes:

No:

1. If yes, how much time did this take you in a week? *(Or, how many times a week did you need to collect fuel?)*
2. Were other members of the household involved in fetching and maybe preparing fuel for the cooking?

*(***Probe:** *preparing wood by chopping into smaller pieces)*

Yes:

No:

1. If yes, for how much time were they involved in a week? *(And how many times a week?)*
2. How much money did you use to spend on fuel?

*(****Probe:*** *follow their lead, they may prefer to answer in terms of days, weeks or months)*

***Now let us switch to the actual cooking of the meals.***

1. Before this study who was involved in the cooking of the food on an average day?
2. For how long was each person (including yourself) involved per day?
3. What other activities, if any, did you use your old method of cooking for? (**Probe:** *was it a source of warmth, light, social space to gather, etc.*)

**After Receiving the Study Cookstove**

*(***Preamble:** *Now I am going to ask you some questions on your experiences AFTER you started using the new CAPS cookstove*)

1. How long do you now spend on cooking each day in total with your study cookstoves?
2. Let us break this down,
   1. in the morning:
   2. in the afternoon:
   3. in the evening:

***Again, let us revisit the time before you even start cooking, when there is the process to fetching and maybe preparing the fuel:***

1. Are you involved in fetching and preparing fuel for the cooking?

Yes:

No:

1. If yes, how much time does this take you in a week? (*How many times a week?)*
2. Are other members of the household involved in fetching fuel and maybe preparing for the cooking?

Yes:

No:

1. If yes, how much time does this take them in a week? (*How many times a week?*)
2. How much money do you now spend on fuel?

***Now let us switch to the actual cooking of the meals.***

1. Who is now involved in the cooking of the food on an average day?
2. How long is each person (including you involved) per day?
3. Do you have more time to do other things now you have this new method of cooking?

Yes:

No:

1. If yes, what other things do you or others in the household do?
2. Have you observed any change the taste of your food?

(**Probe**: *can you explain more, what changes have you noted?*)

1. What is your experience of using the solar panels?
2. We realize the study provides you with 2 cookstoves & a solar panel and helps with their maintenance, but have you had to spend any money on your:

(a) cookstove:

(b) solar panel:

(c) cooking pots and pans and utensils:

(d) other:

1. Where do you do your cooking during the rainy season?
2. Where do you do your cooking during the dry season?
3. Do you like using this cookstove? (Why?)
4. Would you be willing to continue using this stove after CAPS?

Yes:

No:

1. Would you be willing to pay for this stove if it was available in shops?

Yes:

No:

1. How much would you pay?
2. How did you decide on this amount?
3. Are you aware of other stove choices? What are these?
4. Have you used other stove methods? How do they compare to the Philips cookstove?
5. Apart from the benefits mentioned previously, are there any other benefits to the household?
6. Are you aware of any health benefits associated with the cookstoves? Who benefits from this improved health?
7. Do the solar panel, stoves, batteries come in useful in any other ways?
8. We know that you are encouraged to use the cookstoves for all your cooking, but do you also use other cooking methods? If so please explain?
9. Apart from cooking, are you exposed to any other sources of fire or smoke on a daily or almost every day basis?

**(B) VERSION OF THE QUESTIONNAIRE FOR THOSE IN THE INTERVENTION ARM**

**Prior to In-depth interview - Background Information**

**DEMOGRAPHIC DATA**

1. CAPS House Identity Number |__|__|__|__|-|__|__|__|-|__|__|
2. Date of Birth of respondent (dd / mm / yyyy) |__|__|-|__|__|-­|__|__|__|__|
3. Sex (**1**=Male **2**=Female) |__|
4. Husband present during the interview

Yes:

No:

1. How long have you been in the study? |__|__|years |__|__|-months

**SOCIO ECONOMIC DATA**

| 1. Religion 2. Christianity 3. Islam 4. Traditional African Religion 5. None 6. Other___________________ | (cross one) | 1. Marital Status 2. Married 3. In relationship but not married 4. Single 5. Divorced 6. Separated 7. Widowed 8. Other __________________ | *(cross one)* |
| --- | --- | --- | --- |
|  |  | 1. What is your Occupation 2. Housewife 3. Traditional Birth Assistant 4. Nurse/ Midwife 5. Farmer 6. Herding 7. Gardening 8. Sell at the market/street 9. Shopkeeper/ Retail 10. Childcare 11. Domestic Help 12. Student 13. No occupation 14. Other(Specify)___________ | *(cross all that apply)* |
| 1. What is the Occupation of your husband?      1. Imam 2. Marabout 3. Village health worker 4. Farmer 5. Herdsman 6. Fisherman 7. Bricklayer 8. Carpenter 9. Welder 10. Sell at the market/street 11. Shopkeeper/ Retail 12. Retired 13. No Occupation 14. Not applicable 15. Other (Specify) ___________ | *(cross all that apply)* |  |  |
| Do **you own at least 1 of** the following?   1. Radio/cassette 2. Iron bed or carved wood bed 3. Bicycle 4. Horse or donkey cart 5. Boat 6. Video 7. TV 8. Motorbike 9. Watch 10. Mobile phones 11. Car 12. None of the above | *(cross all that apply)*                        On average how much do you spend on air time per week/month? |  |  |
| 1. What is the main source of drinking water in your household 2. Surface water 3. Tanker 4. Well 5. Bore hole 6. Piped water 7. River water 8. Other _____________ | *(cross one)* | 1. What kind of toilet facilities does your household use? 2. No facility 3. Pit latrine 4. Improved pit latrine 5. Flush toilet 6. Other __________________ | *(cross all that apply)* |
| 1. In **your** household does anyone own the following? 2. Goats 3. Sheep 4. Horses 5. Donkey 6. Pigs 7. 0-50 Cattle 8. 51-100 Cattle 9. 101-200 Cattle 10. None of the above | *(cross all that apply)* |  |  |
| 1. What is the main material of the floor? 2. Earth/sand 3. Wooden/bamboo 4. Vinyl/tiles 5. Cement 6. Other _____________________ | (Cross one) | 1. What is the main material of the roof? 2. Plastic/tarpaulin 3. Thatch 4. Iron Sheets or tiles 5. Other ________________ | (cross one) |

Level of education of household head (tick as appropriate)

1. None [ ]
2. Primary [ ]
3. Secondary [ ]
4. Post secondary [ ]

Level of education of household head cook (tick as appropriate)

1. None [ ]
2. Primary [ ]
3. Secondary [ ]
4. Post secondary [ ]

**Introduction to study:**

*Good morning/afternoon, thank you for agreeing to participate in our survey. I’ll be asking you questions on your cooking experiences. We are here to learn from you and none of your answers will affect your place in the study.*

*I will be asking you questions about your experience cooking and your understanding of cookstoves, regarding your time spent collecting fuel, cooking itself, and any impacts cooking has had on your hosehold. Some questions will be yes or no questions, but for others, I encourage you to give as much detail as possible about your experiences.*

**Your current cooking practices**

1. What sort of fuel did you use to prepare meals?

***Before you even start cooking, there is the process to fetching and maybe preparing the fuel:***

1. How do you get your fuel?

*(Preparing means: chopping the wood into smaller pieces, collecting charcoal, etc)*

|  | *Fetching* | *Delivery* | *Preperation of fuel* |
| --- | --- | --- | --- |
| *Yes* |  |  |  |
| *No* |  |  |  |

1. Were you involved in preparing the fuel for the cooking?

*(Preparing means: chopping the wood into smaller pieces, collecting charcoal, etc)*

| *Yes* |
| --- |
| *No* |

1. If yes in Q2 or 3 how much **time** (in hours) does this take you in a week? *(Or, how many times a week did you need to collect fuel?)*

|  | *Fetching* | *Delivery* | *Preperation of fuel* |
| --- | --- | --- | --- |
| *Time in Hours* |  |  |  |

1. Are other members of the household involved in fetching and maybe preparing fuel for the cooking?

*(***Probe:** *preparing wood by chopping into smaller pieces)*

Yes:

No:

1. If yes, for how much time are they involved in a week? *(And how many times a week?)*
2. How much money do you spend on fuel?

*(****Probe:*** *follow their lead, they may prefer to answer in terms of days, weeks or months)*

***Now let us switch to the actual cooking of the meals.***

1. How long did you spend in total on cooking each day:
   1. in the morning:
   2. in the afternoon:
   3. in the evening:
2. What other activities, do you use your cooking method for other than for cooking? (**Probe:** *was it a source of warmth, light, social space to gather, etc.*)
3. Apart from cooking, are you exposed to any other sources of fire or smoke on a daily or almost every day basis?

**What do you know about fan assisted/ advanced Cookstove**

*(***Preamble:** *Now I am going to ask you some questions on your experiences AFTER you started using the new CAPS cookstove*)

1. Can you tell us any benefits you think there are associated with using a fan assisted cookstove?
2. Can you tell us any challenges you think there are associated with using a fan assisted cookstove?
3. Would you be willing to pay for a fan assisted stove if it was available in shops?

Yes:

No:

1. How much would you pay?
2. How did you decide on this amount?
3. Are you aware of the different stove choices? What are these?
4. Have you used other stove methods? How do they compare to your current was of cooking?
5. Are you aware of any health benefits associated with the cookstoves? Who benefits from this improved health?
6. Do you think that the solar panel, stoves, batteries could come in useful in any other ways?
7. Do you have anything else you’d like to mention about methods of cooking or any questions for me?

Thank you very much for your time!

If this data collection tool is used in future, the authors should be made aware and fully acknowledged.
